# Clinical characteristics and prognostic factors of posterior segment intraocular foreign body in a tertiary hospital

**DOI:** 10.1186/s12886-018-1026-5

**Published:** 2019-01-14

**Authors:** Jian Ma, Yao Wang, Li Zhang, Min Chen, Jing Ai, Xiaoyun Fang

**Affiliations:** 1 Eye Center, Second Affiliated Hospital of Zhejiang University School of Medicine, Hangzhou, 310009 China; 2 Zhejiang Provincial Key Lab of Ophthalmology, Hangzhou, China

**Keywords:** Clinical characteristics, Prognostic factors, Intraocular foreign body

## Abstract

**Background:**

To identify the clinical characteristics, prognostic factors and visual outcomes in posterior segment IOFBs patients managed by PPV in a tertiary hospital.

**Methods:**

A retrospective chart review was performed for 56 patients, who had PPV for IOFBs removal between November 2013 and November 2015. The mechanisms of injury, the nature of the IOFBs, the BCVA before and after the surgery, the penetrating site and the complications of the surgery were all collected. Univariate analyses were conducted to evaluate the prognostic factors.

**Results:**

The mean age of the patients was 36.4 years. The nature of IOFBs was mainly metal. Most injuries were commonly caused by hammering the metal. The mean preoperative VA was 2.30 logMAR, and mean final VA was 0.92 logMAR. From univariate analysis, good visual outcome was correlated with the good visual acuity before surgery and poor visual outcome was correlated with the macular break and multiple surgeries.

**Conclusions:**

In a tertiary hospital of eastern China, most cases of IOFBs were work-related. The prognosis of the patients was really well in the patients with good presenting visual acuity. Nevertheless the prognosis was not good for those patients who had macular injury or underwent several surgeries because of retinal detachment, epiretinal membrane or proliferative vitreous retinopathy. Good facilities for eye protection are urgently in demand for the workers indeed.

## Background

Open-globe injury often can result in serious visual loss and afflict most patients in the developing country. IOFBs account for 18–41% of all open-globe injury [1, 2]. Most post-traumatic IOFBs (58–88%) reside in the posterior segment [1–4]. The visual prognosis depends on the IOFB size, the zone of the injury, and the accessible treatment [4–6]. Basically IOFBs should be removed from sclera or sclerocorneal tunnel by PPV. Though more techniques have been applied to remove the IOFBs such as “Magnet Handshake” technique [7] and “Macula Protection by Perfluorocarbon Liquid” [8], the prognosis of the patients was not so good in some areas. China is the biggest developing country in the world. During the past twenty years, thousands of factories have sprung up in the east of China. Due to lack of the protection facilities, a lot of workers got hurt at work. Though the injury of the eye could be found immediately after the accident, not all patients sought the treatment timely. The visual outcomes could be totally different whether the patients would consult the doctor in time or not. With advancement in the microsurgical technique, those severely traumatized eyes can be saved from enucleation. However, some patients received several surgeries and still could not save their sight. The objectives of this study were to identify the clinical characteristics and prognostic factors in posterior segment IOFBs patients managed by 23-gauge PPV in the east of china.

## Methods

The patients who were diagnosed with IOFBs at The Second Affiliated Hospital of Zhejiang University School of Medicine between November 2013 and November 2015 were included for this study. The follow-up ended up with the patients’ last control. The study was approved by Institutional Review Board of Second Affiliated Hospital of Zhejiang University School of Medicine and conducted in compliance with guidelines of the Declaration of Helsinki. The complete history of the patients was taken at their first presentation. The initial BCVA of the patient was recorded using the Snellen chart. It was converted to a logMAR units for statistical analysis. An anterior segment ophthalmologic examination was performed with a slit-lamp (BQ 900; Haag-Streit, Berne,Switzerland). The location of the metallic IOFB was identified by a computerized tomography before operation. The collected data comprised age, gender, mechanism of injury, preoperative VA, initial ocular features, nature of foreign bodies, time interval between injury and IOFBs removal, postoperative VA and complications. All patients with a leaking wound underwent primary wound repair by general ophthalmologists before IOFBs removal surgery. Patients with a self-sealing wound underwent PPV surgery for the initial intervention. A classic three-port, 23-gauge vitrectomy technique was performed by three retinal specialists (J.M., Y.W. and L.Z.). The non-contact wide-angle vitreous surgery system was used during the PPV surgery. The corneal entry site was sutured with Nylon 10–0 and the scleral wound was repaired with Vicryl 8–0. If the view for performing the PPV surgery was obstructed by the traumatic cataract, a lensectomy or phacoemulsification procedure was also done during the surgery. The intraocular lens was not implanted for the first time. IOFBs were removed from the enlarged sclerotomy or limbal incision either with the intraocular forceps or the external magnet. The injured retinal areas, including retinal holes or detached retina were secured by endolaser photocoagulation, cryoretinopexy. Either gas or silicone oil was used for the intraocular tamponade. The patients were regularly controlled by the retinal experts of the Second Affiliated Hospital of Zhejiang University School of Medicine. A PMMA lens with iris fixation was implanted in the anterior chamber or a single-piece PMMA lens was implanted into the ciliary sulcus depending on the integrity of the lens capsule after 3 months. In addition, vitreoretinal operations were performed in case of complications, such as retinal detachment or macular pucker. A good visual outcome was defined as the final BCVA equal to or better than 20/40. A poor visual outcome was determined as final BCVA of less than 20/200.

### Statistical analysis

Statistical analysis of data was performed using SPSS 17.0 (SPSS Inc., Chicago, IL). Continuous data were reported as mean ± standard deviation, and categorical data were reported as n (%). Paired sample *t*-test and chi-square test were used for comparing the preoperative BCVA to the postoperative BCVA. The predictive factors for visual outcomes were studied using univariate analysis (Fisher exact test, or Mann-Whitney U-test). A *P* value of 0.05 was considered statistically significant in this study.

## Results

Of the referred to our center for ocular trauma associated with IOFBs between November 2013 and November 2015, 56 patients were included for this study. The mean follow-up time was 15.6 months (range 9–36, median 13.5, SD 4.7 months) and mean age was 40.8 years (range 8–63, median 40.5, SD 12.9 years). Fifty-four (54/56, 96.4%) patients were male. In all, 54 (54/56, 96.4%) patients were work-related. The nature of the IOFBs was metal in 54 (54/56, 96.4%) patients, glass in 1 (1/56, 1.8%) patient and wood in 1 (1/56, 1.8%) patient. The mechanisms of injury and the nature of the intraocular foreign body are summarized in Table 1 and Table 2. The mean preoperative VA was 2.30 (range 0.00–3.00, median 2.70, SD 0.90) logMAR. The penetrating sites were from cornea (37/56, 66.1%), sclera (11/56, 19.6) and corneosclera (8/56, 14.3%) (Table 3). The preoperative clinical data of the patients are presented in Table 4. More than half of the patients (42/56, 75%) had lens injury. Retinal injury including retinal break, retinal hemorrhage and retinal detachment were seen from 46 patients (46/56, 82.1%). 12 patients (12/56, 21.4%) developed into endophthalmitis because of untreated penetrating site or delayed visiting the doctor. One patient was afflicted by siderosis bulbi (1/56, 1.8%).

**Table 1:** Mechanisms of injury

Mechanism of injury	Number (%)
Hammering the metal	33(58.9)
Using electric drill	5(8.9)
Shaving steel wire	4(7.1)
Air nail gun	3(5.4)
Chiseling on metal	3(5.4)
Looking at others’ working	3(5.4)
Firework explosion	1 (1.8)
Wood cutting	1(1.8)
Stabbed by the pencil	1(1.8)
Hit by the branch	1(1.8)
Electric welding	1(1.8)

**Table 2:** The nature of the intraocular foreign body

Nature of the foreign body	Number(%)
Magnetic Metal	52(92.9)
Gun nail	2(3.6)
Wood	1(1.8)
Glass	1(1.8)

**Table 3:** Penetrating site of the patients

Clinical characteristics	Number (%)
Cornea	37(66.1)
Sclera	11(19.6)
Corneosclera	8(14.3)

**Table 4:** Clinical data of the patients

Clinical characteristics	Number (%)
Hyphema	5(8.9)
Iris injury	2(3.6)
Lens injury	42(75)
Vitreous hemorrhage	20(35.7)
Retinal injury	
Retinal break	20(35.7)
Retinal hemorrhage	19(33.9)
Retinal detachment	7(12.5)
Endophthalmitis	12(21.4)
Siderosis bulbi	1(1.8)

Lens removal was performed with either phacoemulsification or pars plana lensectomy at the time of vitrectomy in 42 (42/56, 75%) patients. Intraocular tamponade was performed at the end of surgery in 52 (52/56, 92.90%) patients. Of these, 25 (25/52, 48.10%) were silicone oil and 27 (27/52, 51.90%) gas tamponade. At the end of follow-up, 4 (4/56, 7.14%) patients had failed to achieve anatomical success with no light perception. The main reasons for this were severe proliferative vitreous retinopathy and macular injury. Anatomical success was achieved in 52 (52/56, 92.90%) patients.

**Fig. 1:**
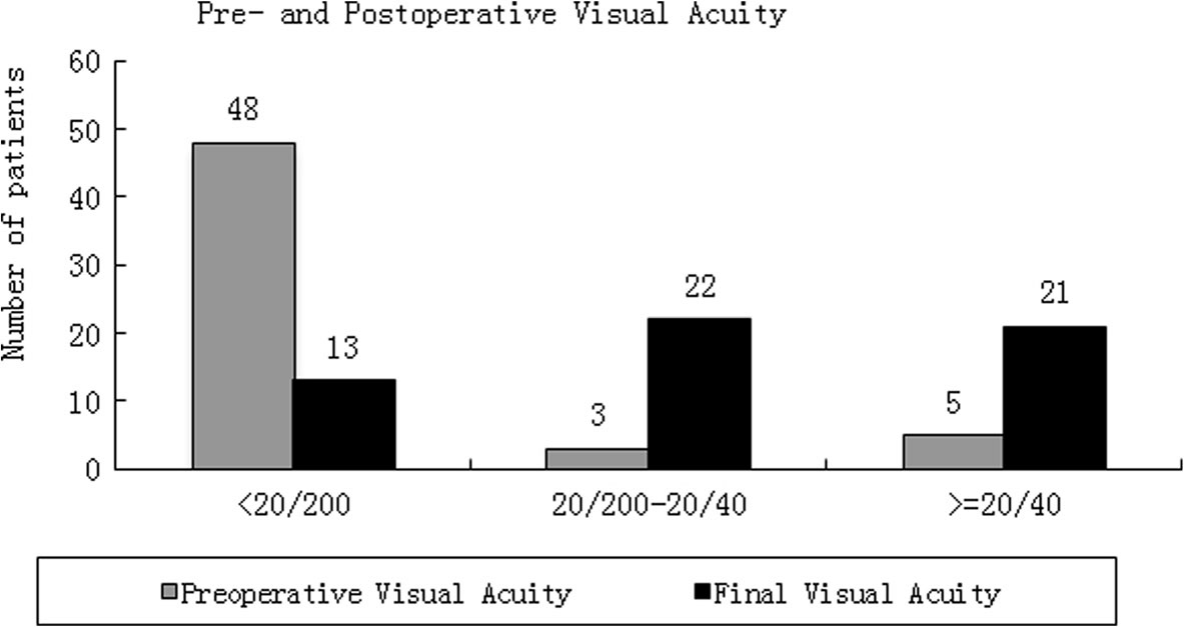
Pre- and Postoperative Visual Acuity. The grey columns showed the pre-operative visual acuity of the patients. The black columns showed the postoperative visual acuity. Almost forty-eight patients had low vision (less than 20/200) before surgery. More than forty-three patients got the good vision after surgery (better than 20/200)

**Fig. 2:**
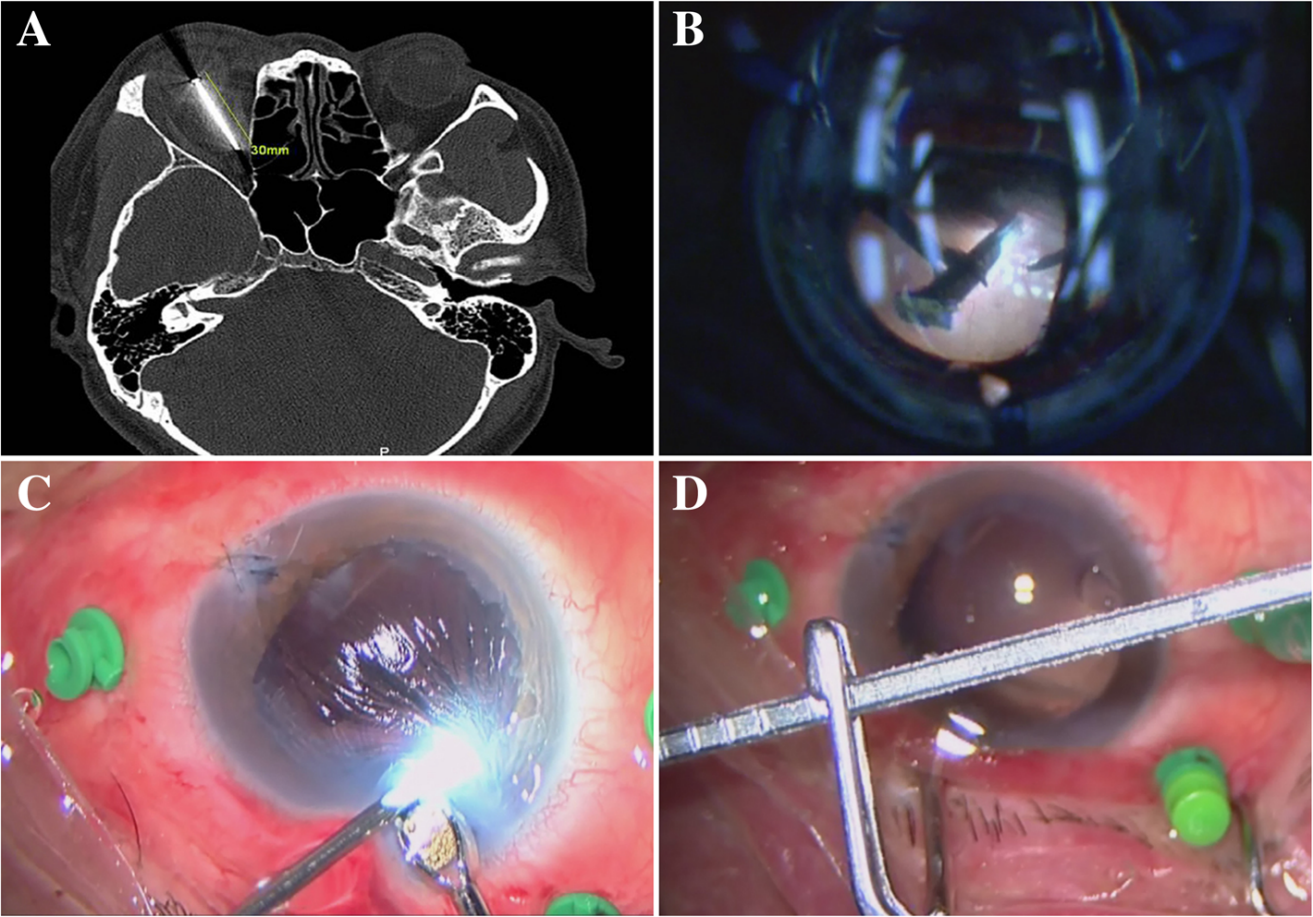
A large IOFB was removed from the limbal incision. **a**: The orbital CT scan of the patient. **b**: Grabbing the IOFB with the intraocular forceps. **c**: Removing the IOFB from the limbal incision. **d**: The whole view of the IOFB in the microscope

The mean final BCVA was 0.92 (range 0.00–3.00, median 0.40, SD 1.03) logMAR. Four (4/56, 7.1%) patients reported no light perception after IOFBs removal. The final BCVA was improved in 44 (44/56, 78.57%) patients, stabilized in 8 (8/56, 14.29%) and worse in 4 (4/56, 7.14%). Preoperative and final visual acuities were demonstrated in Table 5 and Fig. 1. Almost forty-eight (48/56, 85.7%) patients had low vision (less than 20/200) before surgery. More than forty-three (43/56, 76.8%) patients got the good vision (better than 20/200). Postoperative improvement of visual acuity was statistically significant (*P* = 0.008). The large IOFB would be removed from the limbal incision. A 30 mm IOFB was removed from the limbal incision of the patient. The final visual acuity of the patient was surprisingly 20/40 (Fig. 2).

**Table 5:** Preoperative and final visual acuity

VA	Preoperative VA N (%)	Final VA N (%)
> = 20/40	5(8.9)	21(37.5)
< 20/40–20/200	3(5.4)	22(39.3)
< 20/200	48(85.7)	13(23.2)
Total	56(100)	56(100)

Abbreviation: *VA* visual acuity

For good visual outcome, univariate analysis showed that good presenting VA (Fisher exact test, *P* = 0.005) was the significant associated predictors (Table 6). There was no statistical significance for the patients with different entry site, with lens injury, retinal injury and endophthalmitis or not.

**Table 6:** Univariate analysis: predictors for good visual outcome

Predictive factors	Final VA	Final VA	*P*-value^a^
	> = 20/40	< 20/40	
	N (%)	N (%)	
Mean age (years)	38.5	40	0.388
Sex (male: female)	20:1	34:1	0.614
Initial VA > =20/40	5(100)	0	0.005
Entry site			
Cornea	11(29.7)	26(70.3)	0.145
Sclera	5(45.5)	6(54.5)	0.730
Corneosclera	5(62.5)	3(37.5)	0.136
Lens injury	14(33.3)	28(66.7)	0.343
Retinal injury			
Break	6(30)	14(70)	0.565
RD	1(14.3)	6(85.7)	0.237
Hemorrhage	7(36.8)	12(63.2)	0.589
Endophthalmitis	5(41.7)	7(58.3)	0.493

Abbreviations: *RAPD* relative afferent pupillary defect, *RD* rhegmatogenous retinal detachment, *VA* visual acuity

^a^Mann–Whitney U-test; in others Fisher exact test was used

For poor visual outcome, univariate analysis showed that presence of macular break and undergoing multiple vitrectomy (Fisher exact test, *P* = 0.006, 0.0001) were the significant associated predictors (Table 7). No statistical significance was seen from the patients with different initial VA and entry site, with lens injury, retinal injury and endophthalmitis or not.

**Table 7:** Univariate analysis: predictors for poor visual outcome

Predictive factors	Final VA	Final VA	*P*-value
	< 20/200	> = 20/200	
	N (%)	N (%)	
Mean age (years)	39.1	41.3	0.748^a^
Sex (male: female)	13:0	41:2	0.586
Initial VA < 20/200	13(27.1)	35(72.9)	0.349
Entry site			
Cornea	7(18.9)	30(81.1)	0.128
Sclera	4(36.4)	7(63.6)	0.195
Corneosclera	2(25)	6(75)	0.685
Lens injury	8(19)	34(81)	0.080
Retinal injury			
Break	8(40)	12(60)	0.105
RD	4(57.1)	3(42.9)	0.058
Hemorrhage	8(42.1)	11(57.9)	0.051
Endophthalmitis	3(25)	9(75)	0.658
Macular Break	4(100)	0(0)	0.006
Multiple Vitrectomy	7(87.5)	1(12.5)	0.0001

Abbreviations: *RAPD* relative afferent pupillary defect, *RD* rhegmatogenous retinal detachment, *VA* visual acuity. ^a^Mann–Whitney U-test; in others Fisher exact test was used

## Discussion

In the developing country, IOFBs is a serious problem in a young working age population. In accordance with previous reports [5, 9–11], our study showed that the majority of patients (96.4%) were male, with a mean age of 40.8 years. This study found that 91.1% of the patients had work-related injury. Metal was the most common nature of IOFBs, accounting for 96.4% of the patients, which is similar to the other studies [4, 12, 13]. This study also revealed that the hammering the metal was the most common mechanism of injury (58.9%), same as the other study in china [14]. It is different from in Thailand, where the electric grass trimmer was the most common mechanism of injury [15].

There are several tools to remove the IOFBs, including external magnets, intraocular magnets and foreign body claw. Pars plana vitrectomy can be the first choice for the patients with the IOFBs in the posterior segment, though magnetic suction from the sclera is still used in the non-developed areas. With advancement of the surgical facilities and the techniques, 23-gauge vitrectomy was commonly recommended for the posterior IOFBs on account of the less damages and rapid recovery [16]. Sometimes 25-gauge vitrectomy was also used in some cases. However, 25-gauge PPV is not necessary for the patient with large IOFBs. The IOFBs are usually extracted from the corneoscleral limbus or the sclera. It depends on the size of the IOFBs and integrity of the lens. Usually the IOFBs will be removed from the corneoscleral limbus if their diameters are more than 6 mm. For the small ones, the IOFBs can be taken out from the limbus or sclera. We would like to choose the limbus if the patients were diagnosed with traumatic cataract.

We found that good presenting VA before surgery was a significant associated predictor for the good visual outcome. This is the same as the previous reports [10, 17]. But the other study claimed that the initial BCVA was not the best reliable predictive factor for the final BCVA by the multiple correspondence analyses [18].

Poor presenting VA has previously been reported as an important predictive factor for poor visual outcomes [18]. However, our study showed that most patients (35/48) with the initial VA less than 20/200 had better final VA (better than 20/200). It is reasonable that the patients had low presenting VA if the patients suffered traumatic cataract and vitreous hemorrhage. The VA can be improved greatly with cataract extraction and vitreous hemorrhages removal if the macular of the patients remained integrity. Unfortunately the patients had low final VA when they had macular break. Others also reported that the most relevant parameters for a low final BCVA were the presence of a macular lesion [18], RD at presentation and large foreign body [19]. The prognosis was also not good for those patients had several surgeries because of retinal detachment, epiretinal membrane or proliferative vitreous retinopathy. It is due to delayed return visit or a long wait-list for the surgery.

Previous study showed that presence of endophthalmitis, relative afferent pupillary defect (RAPD) and initial RD was the significant associated predictors for the poor final VA [15]. Our study showed the different results. Among 12 patients diagnosed with endophthalmitis, only 3 patients had the low final VA less than 20/200. There is no statistical difference between the final VA less than 20/200 group and the final VA better than 20/200 group. RAPD was not included for this study since some patients had anterior chamber hemorrhages and iris injuries. Initial RD was not the significant predictive factor for the poor final VA in our study. If the detached retina was reattached before the macular was involved, the final VA could be better. Recent study revealed that early removal of IOFB may related to the favourable visual outcome and low endophthalmitis [20]. It means the patient could have a higher chance to recover better if they got the timely treatment.

## Conclusions

In conclusion, most cases of IOFBs were work-related in a tertiary hospital located in the east of china. The prognosis of the patients was really good with good presenting visual acuity. Nevertheless the prognosis was not good for those patients who had macular injury or underwent several surgeries because of retinal detachment, epiretinal membrane or proliferative vitreous retinopathy. Good facilities for eye protection are urgently in demand for the workers indeed.

## Abbreviations

BCVA: Best corrected visual acuity
CT: Computerized tomography
IOFBs: Intraocular foreign bodies
logMAR: Logarithm of the minimal angle of resolution
PPV: Pars plana vitrectomy
VA: Visual acuity
